# Molecular Mechanisms and Clinical Implications of Complex Prehabilitation in Colorectal Cancer Surgery: A Comprehensive Review

**DOI:** 10.3390/ijms26157242

**Published:** 2025-07-26

**Authors:** Jakub Włodarczyk

**Affiliations:** 1 Department of General and Oncological Surgery, Faculty of Medicine, Medical University of Łódź, Pomorska 251, 92-213 Łódź, Poland; jakub.wlodarczyk@umed.lodz.pl; 2 Department of Surgery, Johns Hopkins Medicine, Baltimore, MD 21205, USA

**Keywords:** colorectal cancer, prehabilitation, nutrition, psychological intervention, physical intervention

## Abstract

Colorectal cancer (CRC) remains a leading cause of cancer morbidity and mortality worldwide, especially in older adults where frailty complicates treatment outcomes. Multimodal prehabilitation—comprising nutritional support, physical exercise, and psychological interventions—has emerged as a promising strategy to enhance patients’ resilience before CRC surgery. Clinical studies demonstrate that prehabilitation significantly reduces postoperative complications, shortens hospital stays, and improves functional recovery. Nutritional interventions focus on counteracting malnutrition and sarcopenia through tailored dietary counseling, protein supplementation, and immunonutrients like arginine and glutamine. Physical exercise enhances cardiorespiratory fitness and muscle strength while modulating immune and metabolic pathways critical for surgical recovery. Psychological support reduces anxiety and depression, promoting mental resilience that correlates with better postoperative outcomes. Despite clear clinical benefits, the molecular mechanisms underlying prehabilitation’s effects—such as inflammation modulation, immune activation, and metabolic rewiring—remain poorly understood. This review addresses this knowledge gap by exploring potential biological pathways influenced by prehabilitation, aiming to guide more targeted, personalized approaches in CRC patient management. Advancing molecular insights may optimize prehabilitation protocols and improve survival and quality of life for CRC patients undergoing surgery.

## 1. Introduction

The latest forecasts from the American Cancer Society reveal that colorectal cancer (CRC) still plays a major role in the overall cancer burden nationally and worldwide. Colorectal cancer consistently makes up about 10% of all newly diagnosed cancer cases and roughly 9% of all cancer-related fatalities in the United States [1]. Current estimates suggest that around 151,030 new cases of CRC are identified each year, resulting in approximately 52,580 deaths linked to the illness. These statistics illustrate the significant impact of CRC on public health.

Typically, CRC is more prevalent in individuals aged 50 and older, with the risk increasing with age. Additionally, frailty is a common concern in older cancer patients, as it can affect treatment outcomes and overall recovery [2]. Understanding the interplay between age, frailty, and cancer treatment is crucial for developing effective management strategies that can improve survival rates and quality of life for older adults facing CRC.

### 1.1. Prehabilitation

To augment the physical and psychological well-being of individuals prior to oncological interventions, while concurrently mitigating the incidence of postoperative complications, prehabilitation programs are increasingly acknowledged as a critical component of patient management [3]. Prehabilitation facilitates the preparation of patients for significant surgical procedures through the enhancement of their physical, nutritional, and psychological health prior to the intervention [4]. A conventional prehabilitation program comprises multiple interrelated components, with nutritional counseling serving as a pivotal element designed to improve the patient’s dietary health. Such interventions can bolster immune function and promote wound healing; timely modifications to dietary practices have been demonstrated to decrease morbidity and mortality rates among individuals diagnosed with cancer [5]. Subsequent to nutritional interventions, physical exercise frequently emerges as a central component, concentrating on the enhancement of cardiovascular fitness and muscular strength. Empirical studies suggest that structured exercise regimens, particularly those that are meticulously supervised and sufficiently intense, can substantially elevate aerobic capacity and muscular strength [6]. The psychological dimension of prehabilitation is of paramount importance in preparing patients for CRC surgery [7]. Addressing mental health concerns is imperative, as anxiety and depression can profoundly affect treatment efficacy and recovery trajectories. Prehabilitation programs frequently integrate psychological support mechanisms, encompassing counseling and stress reduction strategies, to assist patients in managing the emotional difficulties associated with a cancer diagnosis and forthcoming surgical procedures [8]. By cultivating a constructive mindset and fostering emotional resilience, these programs can enhance patients’ overall well-being, thereby improving adherence to prehabilitation protocols and ultimately contributing to more favorable surgical outcomes and a higher quality of life following treatment.

In most up to date studies, multimodal prehabilitation in patients undergoing CRC surgery has demonstrated meaningful improvements in clinical outcomes. The PREHAB randomized clinical trial (N = 251) found that patients who received a 4-week supervised prehabilitation program experienced significantly fewer severe postoperative complications compared to standard care (17.1% vs. 29.7%; Odds Ratio (OR) 0.47, 95% Confidential Interval (CI) 0.26–0.87; *p* = 0.02) [9]. According to their study protocol, prehabilitated patients received a standard dosage of 30g of high-quality (whey and casein) protein immediately after exercise and before sleep. Medical complications, particularly respiratory, were also lower in the prehabilitation group (15.4% vs. 27.3%; OR 0.48, 95% CI 0.26–0.89; *p* = 0.02). While improvement in postoperative walking capacity (6 min walking distance) was not statistically significant (mean difference 15.6 m; 95% CI −1.4 to 32.6; *p* = 0.07), secondary measures of functional recovery generally favored prehabilitation. In a Dutch retrospective cohort study (N = 586), patients who underwent prehabilitation (N = 196) had significantly lower overall complication rates (31% vs. 40%, *p* = 0.04) and severe complication rates (20% vs. 31%, *p* = 0.01) compared to those receiving standard care (n = 390) [10]. Additionally, length of hospital stay was reduced (mean 5.80 vs. 6.71 days), and prehabilitation led to net hospital cost savings of EUR 140 per patient (EUR 1109 savings vs. EUR 969 investment). In a Dutch study, prehabilitated patients were given dietary advice in order to achieve adequate oral protein intake spread properly across meals (1.5–1.8 g/kg). Moreover, participants received high-quality protein supplements containing 30 g of whey protein following exercise and before sleep.

A systematic review and meta-analysis including 23 randomized controlled trials (N = 2475) reinforced these findings [11]. Preoperative nutritional interventions significantly reduced postoperative infectious complications (Risk Ratio (RR) 0.65; 95% CI 0.45–0.94) and showed low-quality evidence for reducing length of hospital stay (mean difference −0.87 days; 95% CI −1.58 to −0.17).

### 1.2. Molecular Insight

Clinical studies consistently demonstrate that prehabilitation can enhance nutritional status, boost physical performance, reduce postoperative morbidity, and shorten hospital stays.

However, the vast majority of the existing literature predominantly focuses on clinical endpoints, offering little to no explicit explanation of the underlying molecular or cellular mechanisms that could justify these observed benefits. While the effectiveness of prehabilitation is well documented in terms of functional and physiological outcomes, the biological processes it may modulate—such as inflammation, immune activation, muscle protein synthesis, metabolic rewiring, and tumor microenvironment modulation—remain largely speculative and underexplored.

**Figure 1:**
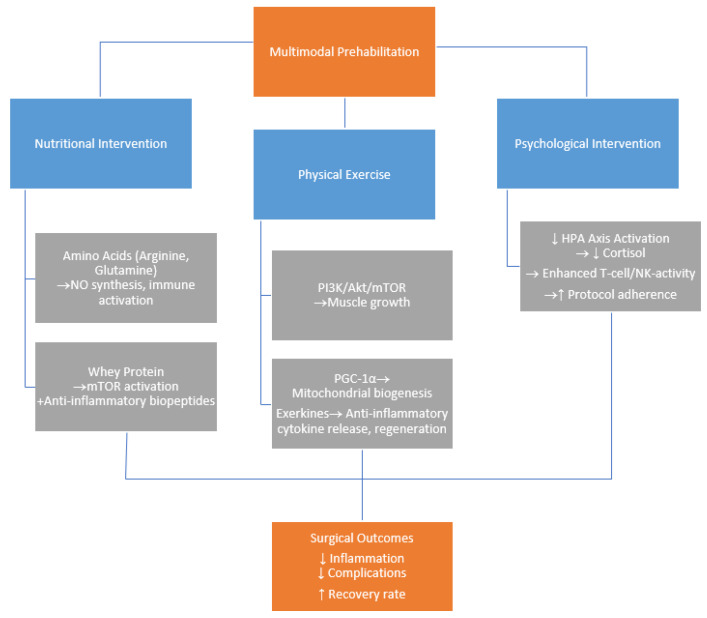
Molecular mechanisms of multimodal prehabilitation in colorectal cancer surgery.

This review aims to bridge this critical knowledge gap by examining the potential molecular pathways influenced by prehabilitation interventions in colorectal cancer patients. Understanding these mechanisms is crucial, as it could facilitate more targeted, personalized, and mechanistically driven prehabilitation strategies in oncological surgery. Figure 1 provides a visual diagram summarizing the molecular mechanisms of multimodal prehabilitation in colorectal cancer surgery.

To ensure a comprehensive synthesis, studies included in this review were identified through targeted literature searches using databases such as PubMed, Scopus, and Web of Science. Keywords and MeSH terms included combinations of “prehabilitation”, “colorectal cancer”, “surgery”, “molecular mechanisms”, “nutrition”, “exercise”, and “psychological intervention”. Only English-language articles published between 2010 and 2025 were considered. Studies that lacked molecular or clinical relevance to multimodal prehabilitation were excluded. Reference lists of key articles were also screened for additional sources.

## 2. The Role of Nutritional Interventions in Prehabilitation

Current academic discussions list various strategies intended to improve nutritional health, which is crucial given the high prevalence of malnutrition and sarcopenia in colorectal cancer patients [12]. Multiple studies highlight the importance of nutritional evaluation; however, its implementation often lacks consistency and is marked by a shortage of reliable assessment tools. The most commonly listed nutritional intervention is individual counseling aimed at guiding patients towards food choices that support their recovery [13]. Unfortunately, a vast number of studies lack a specific description regarding the implemented nutritional intervention. A considerable number of CRC patients experience malnutrition due to increased metabolic demands and reduced appetite. Prehabilitation programs aim to ensure patients receive sufficient caloric intake through personalized meal plans and thorough dietary support, which are crucial for preventing weight loss and maintaining energy levels. Nutritional strategies typically include whey protein supplementation to help meet protein needs and reduce muscle wasting linked to cancer and its treatments [14].

Nutritional interventions are also crucial to counter the other effects of cancer treatment and cancer itself. CRC and its related therapies can lead to shortages of essential vitamins and minerals, such as Vitamin D, calcium, and iron [15]. While the specific scenarios mentioned do not focus on these micronutrients, the general strategy for improving nutritional health usually involves ensuring adequate consumption of these vital nutrients to enhance immune function and overall well-being. The nutritional interventions implemented to address micronutrient deficiencies included the administration of tailored Vitamin D supplements based on age and gender, as well as multivitamin supplements alongside omega-3 fatty acids [16,17].

Immunomodulation through the supplementation of amino acids such as arginine and glutamine has emerged as a promising strategy in the context of nutritional prehabilitation for colorectal cancer patients [18]. Arginine plays a pivotal role in enhancing immune function while glutamine is essential for maintaining the integrity of the intestinal mucosa and supporting the proliferation of lymphocytes, which are crucial for an effective immune response [19,20]. By integrating arginine and glutamine into prehabilitation programs, clinicians can potentially bolster the immune system of colorectal cancer patients, thereby improving their ability to withstand surgical interventions and recover more effectively, while also addressing the nutritional deficits often experienced in this population.

### 2.1. Whey Protein

Whey protein, derived from dairy products, is highly regarded for its exceptional nutritional quality. It contains key proteins such as β-lactoglobulin, α-lactalbumin, lactoferrin, immunoglobulins, and serum albumin, along with bioactive peptides known for their antioxidant and anti-inflammatory properties [21]. These components contribute to whey protein’s effectiveness in combating malnutrition and sarcopenia by promoting muscle protein synthesis, primarily through leucine’s activation of the mammalian target of rapamycin (mTOR) pathway, a central regulator of muscle growth and repair. Its rapid digestion and high bioavailability allow essential amino acids to be quickly absorbed into the bloodstream, enhancing anabolic effects on muscle tissue. α-Lactalbumin (αLA) exhibits anti-inflammatory activity primarily by inhibiting cyclooxygenase-2 (COX-2) and phospholipase A2, which are key enzymes in the inflammatory process [22]. This inhibition reduces the production of pro-inflammatory mediators such as prostaglandin E2 (PGE2) and interleukin-6 (IL-6) in animal models, suggesting its potential as a natural anti-inflammatory agent in dietary sources. Lactoferrin (LF), another milk-derived protein, exerts its anti-inflammatory effects by downregulating the secretion of pro-inflammatory cytokines like tumor necrosis factor- α (TNF-α), IL-1β, IL-6, and IL-8 [23]. This is achieved through interference with the nuclear factor kappa B (NF-κB) pathway, a critical regulator of inflammation. While specific mechanisms for β-lactoglobulin and serum albumin are less detailed in the provided contexts, the general role of food-derived bioactive peptides, including those from these proteins, involves modulation of inflammatory pathways such as NF-κB and mitogen-activated protein kinase (MAPK), as well as interactions with gut microbiota, which are crucial in managing chronic inflammation.

Beyond its benefits in muscle maintenance, whey protein has shown promise in colorectal cancer (CRC) prevention and post-surgical recovery. Animal studies have demonstrated that whey protein hydrolysates can reduce tumor formation and circulating markers associated with cancer growth, likely due to their ability to modulate metabolic functions and strengthen nonspecific immune defenses [24,25,26]. In surgical contexts, particularly for CRC patients, whey protein serves as a supportive nutritional strategy during the perioperative period, aiding in recovery by improving nutritional status and supporting muscle regeneration. Preoperative supplementation has been linked to enhanced functional recovery, including better mobility outcomes [27]. Moreover, whey protein may help alleviate the side effects of chemotherapy, supporting patient well-being and quality of life. Primarily, whey protein helps overcome malnutrition due to chemotherapy-induced side effects such as nausea and vomiting [28]. In animal models, diets containing whey protein have been effective in reducing intestinal mucositis, a common side effect of chemotherapy, further supporting its role in improving the nutritional outcome during treatment [29]. Its natural, cost-effective profile makes it a practical intervention for both cancer prevention and perioperative care. Supplementation has also been associated with improved physical function, including enhanced walking ability, which underscores its role in preserving muscle mass and overall health [30].

In terms of dosing, while no precise guidelines exist for whey protein use specifically in CRC or surgical recovery, general protein intake recommendations serve as a helpful framework. For healthy adults, the recommended dietary allowance (RDA) is about 0.8 g per kilogram of body weight (BW) per day [31]. However, for cancer patients or those recovering from surgery, higher intakes—ranging from 1.2 to 1.5 g/kg BW/day, and up to 2.0 g/kg BW/day in some cases—are often advised, assuming normal kidney and liver function [4]. Ultimately, protein supplementation, including whey protein, should be personalized based on an individual’s medical condition, nutritional needs, and recovery goals.

### 2.2. Molecular Mechanism of Action of Arginine

Arginine is a semi-essential amino acid that plays a central role in several metabolic and cellular pathways involved in the immune system, tissue repair, and vascular regulation—processes particularly relevant in the context of prehabilitation for colorectal cancer patients [32]. Beyond its structural role, arginine serves as a versatile precursor for various enzymes. A key metabolic route involves its conversion by nitric oxide synthase (NOS) enzymes into nitric oxide (NO), a potent vasodilator and signaling molecule that enhances blood flow, modulates vascular tone, and participates in the immune response by aiding in pathogen clearance [33,34,35]. This vasodilatory effect is particularly beneficial in the context of wound healing, where NO regulates collagen formation, cell proliferation, and wound contraction, essential processes in the healing cascade [36]. The administration of L-arginine has been shown to improve cardiovascular function and reduce tissue injury by restoring blood flow and attenuating inflammatory responses [37].

Furthermore, NO’s role in wound healing extends to its involvement in angiogenesis and vasculogenesis, processes critical for the formation of new blood vessels and the repair of damaged tissues [38].

Additionally, arginine donates functional groups in the synthesis of creatine, a molecule that buffers cellular energy, particularly in metabolically active tissues such as skeletal muscle and activated immune cells [39].

### 2.3. Molecular Mechanism of Action of Glutamine

Glutamine is the most abundant free amino acid in the human body and serves multiple functions that extend far beyond its role as a protein building block. Structurally, glutamine possesses two amino groups—one in the main backbone and one in the side chain—allowing it to effectively transport nitrogen between tissues [40,41,42]. Synthesized primarily in skeletal muscle and the liver via the enzyme glutamine synthetase, glutamine acts as a key nitrogen donor and metabolic intermediary. It is catabolized through glutaminolysis by glutaminase (GLS), producing glutamate and ammonia [43,44]. The resulting glutamate can be further converted to α-ketoglutarate, feeding into the Krebs (TCA) cycle to support adenosine triphosphate (ATP) production and biosynthesis of nucleotides and other macromolecules. During physiological stress such as infection, surgery, or chemotherapy, glutamine demand increases dramatically. Adequate glutamine availability ensures effective immune surveillance, while deficiency may impair cytokine signaling and immune cell expansion [20,45,46].

These pathways make glutamine a vital energy source for rapidly proliferating cells, particularly lymphocytes and enterocytes, both of which are highly active during immune responses and tissue repair [20,47].

Glutamine supports immune function through multiple mechanisms: it fuels lymphocyte proliferation, provides precursors for antioxidant glutathione synthesis, and activates intracellular signaling cascades such as the extracellular signal-regulated kinases (ERKs), the c-Jun N-terminal kinases (JNKs), and NF-κB that regulate cytokine production and cell surface marker expression (e.g., cluster of differentiation (CD) 25, CD45RO, and CD71) [41,48,49,50].

Glutamine metabolism affects the polarization of macrophages, which can occur in two main states: M1 and M2. M1 macrophages are pro-inflammatory and are typically induced by lipopolysaccharide (LPS) and interferon-gamma (IFN-γ), while M2 macrophages are anti-inflammatory and are induced by interleukin-4 (IL-4) and interleukin-13 (IL-13) [51]. M2 macrophages consume more glutamine than M1 macrophages, and this glutamine metabolism is essential for their function and polarization. The nuclear receptor peroxisome proliferator-activated receptor gamma (PPARγ) is involved in the glutamine-mediated alternative activation of macrophages [52,53].

Glutamine is also vital for T cell function. It regulates T cell proliferation and activation, with different T cell types having varying requirements for glutamine [54]. Effector T cells, which require rapid proliferation, have a higher rate of glutamine metabolism compared to initial T cells, which only need it for survival [55]. The absence of the glutamine transporter *SLC7A5* inhibits cluster of differentiation (CD) 8 T cell proliferation and affects the mTORC1 signaling pathway, which plays a key role in T cell activation [56,57].

In the tumor microenvironment (TME), glutamine levels influence the function of NK cells and other immune cells. NK cells require an appropriate concentration of glutamine to secrete cytokines like IFN-γ and TNF-α, which are essential for their tumor-killing activity [58]. Additionally, glutamine affects the differentiation and function of B cells, dendritic cells, and myeloid-derived suppressor cells (MDSCs), highlighting its broad role in immune regulation [59,60,61].

In the gut, glutamine is the primary energy source for enterocytes and maintains the intestinal barrier integrity. It promotes protein synthesis, reduces proteolysis, stimulates cellular repair via ERK/JNK pathways, and prevents bacterial translocation by maintaining tight junction proteins [62,63]. Furthermore, glutamine modulates inflammation by regulating the expression of pro-inflammatory cytokines such as TNF-α, IL-6, and IFN-γ through transcriptional control mechanisms [64].

Clinically, glutamine supplementation has shown benefits in CRC patients, particularly in mitigating the gastrointestinal side effects of chemotherapy (e.g., mucositis and malabsorption) and enhancing recovery after colorectal surgery [65,66,67]. By improving gut barrier function and modulating immune responses, glutamine supplementation has been associated with reduced infection rates, improved nutrient absorption, and shorter hospital stays, thereby contributing to better overall outcomes during perioperative care [68,69]. In patients receiving total parenteral nutrition (TPN), glutamine dipeptides are often preferred due to their stability and compatibility with fluid-restricted regimens. These multifaceted roles underscore glutamine’s critical contribution to metabolic resilience and immune competence in the prehabilitation of colorectal cancer patients.

### 2.4. Molecular Mechanism of Action of Omega-3

Omega-3 polyunsaturated fatty acids (PUFAs), particularly eicosapentaenoic acid (EPA) and docosahexaenoic acid (DHA), exert multifaceted biochemical and immunomodulatory effects that seem to play a relevant role in the context of surgical stress and cancer-related inflammation [70]. At the cellular level, these fatty acids are incorporated into the phospholipid bilayer of immune cell membranes, altering membrane fluidity, flexibility, and lipid raft composition. Such changes affect membrane protein distribution and receptor-mediated signaling, particularly in lymphocytes, macrophages, and dendritic cells, thereby modulating cell activation, antigen presentation, and cytokine production [71,72,73,74]. Through the remodeling of lipid rafts—cholesterol- and sphingolipid-enriched microdomains that facilitate signaling—PUFAs disrupt receptor clustering and impair downstream pro-inflammatory signaling from Toll-like receptors (TLRs), attenuating MAPK and protein kinase C (PKC) activation [75,76,77].

At the molecular level, PUFAs suppress inflammation by modulating gene expression via key transcriptional regulators [70]. EPA and DHA inhibit the nuclear translocation and transcriptional activity of NF-κB, a master regulator of inflammatory gene expression, resulting in decreased production of pro-inflammatory cytokines such as TNF-α, IL-6, and IL-1β [77,78,79]. Simultaneously, omega-3 fatty acids activate peroxisome proliferator-activated receptor gamma (PPAR-γ), a nuclear receptor that downregulates inflammatory gene transcription and further antagonizes NF-κB activity [80]. Additionally, omega-3 fatty acids serve as precursors to specialized pro-resolving mediators (SPMs), including resolvins and protectins, which actively promote the resolution of inflammation and facilitate tissue repair [81,82].

On the immune cellular level, omega-3 fatty acids exert targeted effects by modulating the function and phenotype of key immune populations. They inhibit excessive T cell proliferation and reduce hyperinflammatory responses in both T and B cells, contributing to controlled adaptive immune activity [83,84,85]. Furthermore, they promote macrophage polarization toward the M2 phenotype, which is characterized by anti-inflammatory, pro-resolving, and tissue-reparative functions—a shift crucial for mitigating postoperative inflammation and promoting healing [86]. Omega-3 fatty acids also engage G-protein coupled receptors such as GPR120, which transduce anti-inflammatory signals and further downregulate cytokine production [79,87,88].

### 2.5. Molecular Mechanism of Action of Vitamin D

Vitamin D plays a molecular and immunological role in maintaining gastrointestinal health and modulating inflammatory responses, making it highly relevant in the prehabilitation of colorectal cancer patients [89,90]. Functioning as a secosteroid hormone, the active form of vitamin D—1,25-dihydroxyvitamin D_3_—exerts its effects primarily through the vitamin D receptor (VDR), a nuclear receptor expressed in numerous cell types, including epithelial and immune cells. Upon ligand binding, the VDR heterodimerizes with the retinoid X receptor (RXR) and binds to vitamin D response elements (VDREs) in the promoter regions of target genes, regulating the transcription of a wide array of genes involved in immune modulation, cell differentiation, and epithelial integrity.

On a molecular level, vitamin D suppresses pro-inflammatory signaling by inhibiting key pathways such as NF-κB and MAPK, thereby downregulating the production of pro-inflammatory cytokines including TNF-α, IL-6, and IL-17 [91,92,93]. Concurrently, it enhances anti-inflammatory cytokine expression, particularly IL-10, facilitating a shift toward immune tolerance [94]. This immunomodulatory effect spans both innate and adaptive immunity: vitamin D reduces the maturation and antigen-presenting capacity of dendritic cells, inhibits Th1 and Th17 responses, and promotes regulatory T cell (Treg) differentiation [95,96]. It also modulates B cell proliferation and antibody production, contributing to an overall reduction in immune hyperactivation and autoimmune potential [97,98].

Biochemically, vitamin D is a significant factor in maintaining the structural and functional integrity of the intestinal epithelium [89,99]. It enhances tight junction protein expression (e.g., claudins and occludins), thereby strengthening the intestinal barrier and reducing permeability—a crucial factor in preventing bacterial translocation and systemic inflammation [100,101]. Additionally, vitamin D influences gut microbiota composition, promoting microbial diversity and reducing the prevalence of pathogenic bacteria, which together support mucosal homeostasis [102,103].

Adequate vitamin D levels have been linked to enhanced mucosal healing, reduced infection risk—including from *Clostridium difficile*—and improved overall outcomes [104,105,106]. In the context of prehabilitation for CRC surgery, ensuring sufficient vitamin D status may optimize immune resilience, maintain gut barrier integrity, and potentially improve postoperative recovery and reduce complications.

## 3. The Role of Physical Interventions in Prehabilitation

Physical exercise is a central component of multimodal prehabilitation programs. Beyond improving cardiorespiratory fitness and muscle strength, exercise functions as a potent biological modulator, influencing key molecular, biochemical, and immune pathways that contribute to enhanced surgical resilience, reduced postoperative complications, and accelerated recovery [107,108,109]. The primary objective of physical prehabilitation is not merely to preserve baseline functional status, but to proactively build physiological reserve, thereby enabling patients to better withstand the catabolic challenges of surgery and to recover more effectively.

### 3.1. Molecular and Biochemical Adaptations to Exercise

Physical training, especially when combining aerobic exercise (AE) and resistance exercise (RE), induces a cascade of molecular events that enhance skeletal muscle anabolism, mitochondrial efficiency, and overall metabolic flexibility [110,111,112]. Resistance training activates the PI3K/Akt/mTOR signaling axis, which promotes protein synthesis, muscle fiber hypertrophy, and preservation of lean body mass—crucial adaptations for counteracting the muscle catabolism associated with surgical stress and malnutrition [113,114,115]. Concurrently, AE upregulates peroxisome proliferator-activated receptor gamma coactivator-1 alpha (PGC-1α), a key regulator of mitochondrial biogenesis and oxidative metabolism, improving energy utilization and fatigue resistance during recovery [116]. A pivotal mechanism underpinning these adaptations involves the release of exerkines, a class of exercise-induced bioactive molecules that includes myokines, adipokines, cardiokines, and hepatokines [117,118]. These molecules function via endocrine, paracrine, and autocrine signaling to coordinate systemic metabolic responses. Among the most well-studied exerkines, follistatin, irisin, IL-6, and insulin-like growth factor-1 (IGF-1) play crucial roles in modulating inflammation, muscle regeneration, and metabolic homeostasis.

Follistatin inhibits myostatin, a potent negative regulator of muscle growth, thereby promoting muscle preservation and hypertrophy [119,120]. Irisin enhances energy expenditure by inducing browning of white adipose tissue, which improves metabolic flexibility [121]. IGF-1, stimulated by resistance exercise, augments protein synthesis and satellite cell activation, further supporting muscle repair [122,123]. IL-6, though traditionally categorized as a pro-inflammatory cytokine, exhibits context-dependent effects; acute exercise-induced IL-6 acts as a myokine that promotes anti-inflammatory signaling and improves glucose and lipid metabolism [124]. Moreover, repeated bouts of moderate-intensity physical activity promote a systemic anti-inflammatory environment, partly through the transient elevation of IL-6, which paradoxically induces the production of anti-inflammatory cytokines such as IL-10 and suppresses pro-inflammatory mediators including TNF-α and IL-1β [125,126].

Furthermore, exercise influences immune cell composition and function. It increases the circulation and cytotoxic activity of natural killer (NK) cells, supports the expansion of regulatory T cells (Tregs), and facilitates the polarization of macrophages toward the M2 phenotype, which is associated with tissue repair and the resolution of inflammation [127,128,129]. These changes enhance wound healing, reduce infection rates, and may lower the risk of surgical complications.

These molecular adaptations are particularly relevant in the setting of cancer cachexia, a condition marked by systemic inflammation, muscle wasting, and metabolic dysregulation [130]. Exercise-mediated reductions in circulating myostatin, along with increased expression of anabolic and anti-inflammatory exerkines, may counteract catabolic processes and improve physical function and quality of life in colorectal cancer patients, particularly those undergoing surgery [117,131,132].

### 3.2. Physiological and Functional Improvements

From a physiological perspective, AE improves cardiopulmonary fitness, measured by peak oxygen uptake (VO_2_ peak), which correlates with postoperative outcomes and is especially relevant in surgeries requiring prolonged anesthesia [133]. Enhanced oxygen transport and utilization capacity supports tissue perfusion and reduces the risk of hypoxia-related complications postoperatively [134]. On the other hand, resistance training directly targets skeletal muscle strength and mass—parameters strongly associated with functional independence and reduced hospital stays [135,136,137,138,139,140].

Several randomized controlled trials have demonstrated that multimodal prehabilitation programs incorporating both AE and RE lead to significant improvements in functional assessments, including hand grip strength, the six-minute walk test (6MWT), and the timed-up-and-go (TUG) test [109,141,142]. These gains are particularly meaningful in colorectal cancer patients, many of whom are at risk for sarcopenia and reduced physical reserve due to systemic inflammation, poor nutritional status, and tumor burden.

Notably, exercise amplifies the effectiveness of nutritional prehabilitation by enhancing nutrient utilization and promoting anabolism [143,144,145]. When combined with high-protein diets or amino acid supplementation, exercise synergistically increases muscle protein accretion, preserves lean body mass, and attenuates muscle atrophy. This combination approach has been shown to improve postoperative mobility, reduce fatigue, and shorten recovery times.

## 4. The Role of Psychological Interventions in Prehabilitation

Psychological interventions play an increasingly recognized role in the multidisciplinary approach to prehabilitation. The integration of psychological support within prehabilitation aims to optimize mental resilience, reduce psychological morbidity, and enhance overall physiological readiness for surgery [146,147]. These interventions, typically designed to reduce anxiety and depression and to enhance self-efficacy and adaptive coping, have demonstrated measurable clinical benefits in surgical outcomes and may also influence recovery through underlying molecular and immunological pathways.

### 4.1. Clinical Impact of Psychological Prehabilitation

Psychological well-being in the preoperative period has consistently been associated with improved postoperative outcomes across a variety of surgical contexts, including colorectal procedures [148,149]. Elevated levels of preoperative anxiety and depression correlate with longer hospital stays, increased postoperative pain, greater complication rates, and delayed recovery [150,151]. Conversely, higher preoperative self-efficacy—the belief in one’s capacity to influence health outcomes—has been associated with faster return of function, reduced analgesic needs, and improved postoperative satisfaction.

Structured psychological interventions, often delivered as part of a trimodal prehabilitation approach alongside physical and nutritional optimization, are increasingly being integrated into patient education and preparation protocols [152]. These programs aim to alleviate procedural anxiety and provide patients with coping strategies to manage both surgical stress and postoperative rehabilitation. Studies have shown that such interventions not only reduce psychological distress but also enhance compliance with prehabilitation protocols, leading to improved postoperative outcomes and quality of life [153,154,155].

Importantly, psychological factors such as pain catastrophizing, kinesiophobia, and low emotional resilience have been implicated in worse functional outcomes following surgery. For instance, in total hip and knee arthroplasty, as well as in laparoscopic and abdominal procedures, preoperative anxiety and depressive symptoms have been strongly linked with increased postoperative pain and lower functional recovery [156,157]. These findings underscore the clinical imperative to address psychological readiness as part of comprehensive surgical care.

### 4.2. Behavioral Mechanisms and Patient Adherence

A critical mechanism through which psychological interventions exert their benefit is the promotion of behavioral change and patient adherence [158]. Cognitive behavioral therapy (CBT), stress management training, and motivational psychological nursing have demonstrated efficacy in reducing psychological distress, thereby increasing engagement with exercise, nutrition, and physical therapy components of prehabilitation [159].

Improved mental well-being fosters higher levels of self-regulation and motivation, facilitating greater adherence to prehabilitation protocols. Enhanced self-efficacy has been shown to empower patients to actively participate in their recovery, even when confronted with postoperative discomfort or fatigue. These behavioral shifts are vital, as adherence to multimodal prehabilitation programs is directly linked to reductions in opioid consumption, improved pain management, and better surgical outcomes.

Motivational psychological nursing, in particular, has been effective in interventional and oncologic surgical populations, improving both mood and compliance with perioperative care plans. Studies indicate that psychological readiness directly influences rehabilitation participation, especially in colorectal cancer patients, who often face multifactorial recovery challenges due to systemic inflammation, cancer-related fatigue, and treatment-related stress.

### 4.3. Molecular, Biochemical, and Immune Mechanisms

Beyond behavioral and psychological constructs, emerging evidence suggests that psychological interventions may influence surgical recovery via neuroendocrine, molecular, and immunological mechanisms [160,161]. Chronic psychological distress—particularly anxiety and depression—activates the hypothalamic–pituitary–adrenal (HPA) axis, resulting in sustained elevations of cortisol, a glucocorticoid with immunosuppressive and catabolic properties [162,163]. Elevated cortisol levels are known to impair immune cell function, delay wound healing, and promote a systemic pro-inflammatory state [164,165,166].

Psychological prehabilitation may attenuate these effects by downregulating hypothalamic–pituitary–adrenal axis (HPA) axis activity and reducing cortisol levels [162,167]. This, in turn, can lead to a more favorable inflammatory profile, characterized by lower circulating levels of cytokines such as IL-6, TNF-α, and C-reactive protein (CRP)—all of which have been associated with poorer surgical outcomes and delayed recovery in CRC patients [168,169,170].

In addition to direct neuroendocrine effects, psychological interventions may exert indirect immunological benefits via behaviorally mediated pathways. Improved psychological health has been associated with better sleep, enhanced nutritional intake, and higher levels of physical activity, all of which positively influence immune function [171,172,173]. For instance, better regulated sleep–wake cycles and improved stress management have been linked to enhanced T cell activity, reduced neutrophil-to-lymphocyte ratios, and improved overall immune surveillance [174,175,176]. These immune parameters have prognostic value in CRC and may contribute to reduced perioperative morbidity when optimized [177,178,179].

Despite these promising hypotheses, the molecular pathways linking psychological interventions to surgical recovery remain under-investigated. Most current studies emphasize clinical and psychological endpoints without concurrently measuring biochemical or immune markers.

## 5. Challenges and Limitations in Current Research

Current research in the area of molecular effects in prehabilitation in colorectal cancer faces several challenges and limitations, primarily due to the lack of basic science studies of prehabilitation programs and the lack of standardized protocols. The concept of prehabilitation, which aims to enhance patients’ physical and psychological readiness for surgery, is still insufficiently investigated, and its role remains controversial due to the variability in interventions and outcomes across studies [142,180]. For example, certain programs concentrate exclusively on nutritional enhancement, whereas others implement a multimodal strategy that encompasses physical exercise and psychological assistance. The lack of standardized outcome metrics further underlines the challenges associated with evaluating the effectiveness of prehabilitation. Establishing uniform protocols necessitates an agreement among researchers and clinicians regarding the crucial components of prehabilitation. These components should encompass criteria for patient selection, precise nutritional and exercise interventions, and quantifiable outcomes such as functional capacity, complication incidence, and quality of life. Standardization would enable the aggregation of data from multiple studies, facilitating meta-analyses that yield more compelling evidence for the advantages of prehabilitation. Additionally, the absence of randomized trials and the heterogeneity of existing studies preclude definitive conclusions about the efficacy of multimodal prehabilitation, which includes physical training, nutritional, and psychological support [142,181,182].

In the realm of molecular testing, while it offers potential for personalized treatment strategies, the practical application is limited by the reproducibility of biomarkers and the need for routine testing of specific genetic mutations [183]. Overall, the field requires more robust, large-scale trials to establish standardized prehabilitation protocols and to better understand the molecular underpinnings that could guide personalized prehabilitation strategies in colorectal cancer.

While nutritional support is a cornerstone of prehabilitation, certain nutrients—particularly those involved in angiogenesis and cellular proliferation—may pose theoretical oncologic risks. Glutamine, for instance, is not only vital for immune and gut barrier function but also serves as a key metabolic substrate for rapidly dividing tumor cells. Its role in fueling the tricarboxylic acid cycle, nucleotide synthesis, and redox balance has led to the concept of “glutamine addiction” in various malignancies [51,184]. Moreover, glutamine metabolism has been shown to promote angiogenesis, a process essential for tumor growth and metastasis [51,184]. Although preclinical studies suggest that targeting glutamine pathways—such as through glutaminase inhibition—can suppress tumor progression and neovascularization, the clinical relevance of these findings in the context of perioperative supplementation remains uncertain [185]. Similar concerns apply to other immunonutrients like arginine and omega-3 fatty acids, which may influence vascular remodeling and immune modulation. Therefore, while current evidence supports the safety and efficacy of nutritional prehabilitation in colorectal cancer surgery, future studies should carefully evaluate the oncologic implications of specific nutrient formulations, particularly in patients with active or residual disease.

Although this review centers on the surgical implications of prehabilitation, emerging evidence suggests that certain nutritional components may also influence the tumor microenvironment and interact with neoadjuvant therapies. For instance, glutamine potentially affects chemotherapy sensitivity depending on the tumor type and metabolic phenotype [186,187]. Omega-3 fatty acids have demonstrated anti-inflammatory and immunomodulatory effects, including modulation of tumor-associated macrophages and enhancement of chemotherapy efficacy in preclinical models [187,188]. Vitamin D may improve epithelial integrity and immune surveillance, and has been associated with better outcomes in patients receiving chemotherapy. These nutrients may alter stromal composition, cytokine profiles, and immune cell infiltration, thereby shaping the tumor’s response to systemic therapy. While clinical data remain limited, integrating molecular endpoints into future prehabilitation trials could clarify these interactions and enhance oncologic relevance.

Despite the growing interest in multimodal prehabilitation for CRC surgery, several limitations persist in the current body of evidence. First, many of the hypothesized molecular mechanisms—such as modulation of inflammation, immune activation, and metabolic rewiring—are extrapolated from studies conducted in non-oncologic or non-surgical contexts, including animal models and chronic inflammatory conditions. This limits the direct applicability of these findings to CRC surgical patients and underscores the need for mechanistic studies embedded within clinical trials.

Although multimodal prehabilitation has consistently demonstrated benefits in improving postoperative outcomes for CRC patients—including reduced complication rates, shorter hospital stays, and enhanced functional recovery—there remains a lack of standardized, evidence-based guidelines to inform clinical practice. The optimal composition, duration, and intensity of prehabilitation programs are still under investigation, and existing studies vary widely in design, patient selection, and outcome measures. Given this uncertainty, a pragmatic and individualized approach appears most appropriate at present. Specifically, referring CRC patients to a dietitian and physiotherapist prior to surgery allows for tailored nutritional and physical optimization based on baseline assessments, comorbidities, and treatment timelines. This strategy aligns with current best practices and emerging consensus from quality improvement initiatives, while awaiting more definitive guidance from ongoing trials and consensus statements.

Prehabilitation in CRC surgery presents distinct challenges and opportunities compared to other cancer types due to the anatomical, physiological, and treatment-specific characteristics of CRC. Unlike many solid tumors, CRC often involves major abdominal surgery, which carries a high risk of postoperative complications such as ileus, anastomotic leakage, and surgical site infections. These risks are compounded by the frequent presence of malnutrition, sarcopenia, and gut microbiota dysbiosis, making nutritional optimization particularly critical in CRC patients. Moreover, rectal cancer patients frequently undergo neoadjuvant chemoradiotherapy, which can impair physical fitness and immune function prior to surgery—a scenario less common in other cancers. The gut-centric nature of CRC also means that interventions like glutamine supplementation and vitamin D may have more direct effects on intestinal barrier integrity and local immune modulation. Additionally, the short preoperative window typical in CRC surgery (4 weeks) necessitates rapid, targeted prehabilitation strategies that balance efficacy with feasibility. These factors collectively distinguish CRC prehabilitation from protocols used in breast, lung, or prostate cancer, where surgical stressors, nutritional demands, and treatment timelines may differ substantially.

Future research should focus on addressing the gaps in knowledge and practice that currently limit the effectiveness of prehabilitation. There is a growing interest in understanding the molecular mechanisms underlying CRC to develop targeted therapies that can be integrated with prehabilitation efforts. Advances in molecular analyses have identified few possible molecular pathways, which could be targeted to boost treatment efficacy and patient outcomes [144,183]. The integration of molecular insights with prehabilitation could potentially lead to personalized therapeutic strategies that not only improve surgical outcomes but also address treatment-related toxicities and disease progression. Additionally, exploring the integration of prehabilitation into existing surgical pathways and enhanced recovery programs will help establish its role as a standard component of perioperative care. Collaboration among researchers, clinicians, policymakers, and technology developers is essential to advance the field of prehabilitation. By working together, these stakeholders can overcome the challenges of implementation and harness the opportunities offered by innovation. Ultimately, prehabilitation has the potential to transform surgical care, empowering patients and improving outcomes on a global scale.

## References

[1] Siegel R.L., Wagle N.S., Cercek A., Smith R.A., Jemal A. (2023). Colorectal Cancer Statistics, 2023. CA Cancer J. Clin..

[2] Mohile S.G., Dale W., Somerfield M.R., Schonberg M.A., Boyd C.M., Burhenn P.S., Canin B., Cohen H.J., Holmes H.M., Hopkins J.O. (2018). Practical Assessment and Management of Vulnerabilities in Older Patients Receiving Chemotherapy: Asco Guideline for Geriatric Oncology. J. Clin. Oncol..

[3] Garoufalia Z., Emile S.H., Meknarit S., Gefen R., Horesh N., Zhou P., Aeschbacher P., Strassmann V., Wexner S.D. (2024). A Systematic Review and Meta-Analysis of High-Quality Randomized Controlled Trials on the Role of Prehabilitation Programs in Colorectal Surgery. Surgery.

[4] Arends J., Bachmann P., Baracos V., Barthelemy N., Bertz H., Bozzetti F., Fearon K., Hütterer E., Isenring E., Kaasa S. (2017). ESPEN Guidelines on Nutrition in Cancer Patients. Clin. Nutr..

[5] Moran J., Guinan E., McCormick P., Larkin J., Mockler D., Hussey J., Moriarty J., Wilson F. (2016). The Ability of Prehabilitation to Influence Postoperative Outcome after Intra-Abdominal Operation: A Systematic Review and Meta-Analysis. Surgery.

[6] Hughes M.J., Hackney R.J., Lamb P.J., Wigmore S.J., Christopher Deans D.A., Skipworth R.J.E. (2019). Prehabilitation Before Major Abdominal Surgery: A Systematic Review and Meta-Analysis. World J. Surg..

[7] Silver J.K., Baima J., Mayer R.S. (2013). Impairment-Driven Cancer Rehabilitation: An Essential Component of Quality Care and Survivorship. CA Cancer J. Clin..

[8] Carli F., Scheede-Bergdahl C. (2015). Prehabilitation to Enhance Perioperative Care. Anesthesiol. Clin..

[9] Molenaar C.J.L., Minnella E.M., Coca-Martinez M., ten Cate D.W.G., Regis M., Awasthi R., Martínez-Palli G., López-Baamonde M., Sebio-Garcia R., Feo C.V. (2023). Effect of Multimodal Prehabilitation on Reducing Postoperative Complications and Enhancing Functional Capacity Following Colorectal Cancer Surgery: The PREHAB Randomized Clinical Trial. JAMA Surg..

[10] Sabajo C.R., ten Cate D.W.G., Heijmans M.H.M., Koot C.T.G., van Leeuwen L.V.L., Slooter G.D. (2024). Prehabilitation in Colorectal Cancer Surgery Improves Outcome and Reduces Hospital Costs. Eur. J. Surg. Oncol..

[11] Steffens D., Nott F., Koh C., Jiang W., Hirst N., Cole R., Karunaratne S., West M.A., Jack S., Solomon M.J. (2024). Effectiveness of Prehabilitation Modalities on Postoperative Outcomes Following Colorectal Cancer Surgery: A Systematic Review of Randomised Controlled Trials. Ann. Surg. Oncol..

[12] Vergara-Fernandez O., Trejo-Avila M., Salgado-Nesme N. (2020). Sarcopenia in Patients with Colorectal Cancer: A Comprehensive Review. World J. Clin. Cases.

[13] Gillis C., Buhler K., Bresee L., Carli F., Gramlich L., Culos-Reed N., Sajobi T.T., Fenton T.R. (2018). Effects of Nutritional Prehabilitation, With and Without Exercise, on Outcomes of Patients Who Undergo Colorectal Surgery: A Systematic Review and Meta-Analysis. Gastroenterology.

[14] van Exter S.H., Drager L.D., van Asseldonk M.J.M.D., Strijker D., van der Schoot N.D., van den Heuvel B., Verlaan S., van den Berg M.G.A. (2023). Adherence to and Efficacy of the Nutritional Intervention in Multimodal Prehabilitation in Colorectal and Esophageal Cancer Patients. Nutrients.

[15] Song M., Garrett W.S., Chan A.T. (2015). Nutrients, Foods, and Colorectal Cancer Prevention. Gastroenterology.

[16] Jahan I., Islam M.A., Harun-Ur-Rashid M., Sultana G.N.N. (2024). Cancer Prevention at the Microscopic Level with the Potent Power of Micronutrients. Heliyon.

[17] Muscaritoli M., Arends J., Bachmann P., Baracos V., Barthelemy N., Bertz H., Bozzetti F., Hütterer E., Isenring E., Kaasa S. (2021). ESPEN Practical Guideline: Clinical Nutrition in Cancer. Clin. Nutr..

[18] Kaźmierczak-Siedlecka K., Daca A., Folwarski M., Makarewicz W., Lebiedzińska A. (2021). Immunonutritional Support as an Important Part of Multidisciplinary Anti-Cancer Therapy. Cent. Eur. J. Immunol..

[19] Kang K., Shu X.L., Zhong J.X., Yu T.T., Lei T. (2014). Effect of L-Arginine on Immune Function: A Meta-Analysis. Asia Pac. J. Clin. Nutr..

[20] Cruzat V., Rogero M.M., Keane K.N., Curi R., Newsholme P. (2018). Glutamine: Metabolism and Immune Function, Supplementation and Clinical Translation. Nutrients.

[21] Minj S., Anand S. (2020). Whey Proteins and Its Derivatives: Bioactivity, Functionality, and Current Applications. Dairy.

[22] Yamaguchi M., Yoshida K., Uchida M. (2009). Novel Functions of Bovine Milk-Derived Alpha-Lactalbumin: Anti-Nociceptive and Anti-Inflammatory Activity Caused by Inhibiting Cyclooxygenase-2 and Phospholipase A2. Biol. Pharm. Bull..

[23] Majumder K., Mine Y., Wu J. (2016). The Potential of Food Protein-Derived Anti-Inflammatory Peptides against Various Chronic Inflammatory Diseases. J. Sci. Food Agric..

[24] Attaallah W., Yılmaz A.M., Erdoğan N., Yalçın A.S., Aktan A.Ö. (2012). Whey Protein versus Whey Protein Hydrolyzate for the Protection of Azoxymethane and Dextran Sodium Sulfate Induced Colonic Tumors in Rats. Pathol. Oncol. Res..

[25] Liu E., Yang M., Li Q., Cheng Q., Wang Y., Ye L., Tian F., Ding H., Ling Y., Xia M. (2023). Antitumor Activity of a Whey Peptide-Based Enteral Diet in C26 Colon Tumor-Bearing Mice. J. Food Sci..

[26] Cacciola N.A., Venneri T., Salzano A., D’Onofrio N., Martano M., Saggese A., Vinale F., Neglia G., Campanile C., Baccigalupi L. (2023). Chemopreventive Effect of a Milk Whey By-Product Derived from Buffalo (Bubalus Bubalis) in Protecting from Colorectal Carcinogenesis. Cell Commun. Signal..

[27] Feng D., Han D., Li M., Li H., Li N., Liu T., Wang J. (2024). Protein Nutritional Support: The Prevention and Regulation of Colorectal Cancer and Its Mechanism Research. Food Front..

[28] Cereda E., Turri A., Klersy C., Cappello S., Ferrari A., Filippi A.R., Brugnatelli S., Caraccia M., Chiellino S., Borioli V. (2019). Whey Protein Isolate Supplementation Improves Body Composition, Muscle Strength, and Treatment Tolerance in Malnourished Advanced Cancer Patients Undergoing Chemotherapy. Cancer Med..

[29] Boukhettala N., Ibrahim A., Aziz M., Vuichoud J., Saudan K.Y., Blum S., Déchelotte P., Breuillé D., Coëffier M. (2010). A Diet Containing Whey Protein, Free Glutamine, and Transforming Growth Factor-β Ameliorates Nutritional Outcome and Intestinal Mucositis during Repeated Chemotherapeutic Challenges in Rats. J. Nutr..

[30] Zhang L., Liu G., Huang X., He F. (2025). Effects of Protein Supplementation on Muscle Mass, Muscle Strength, and Physical Performance in Older Adults with Physical Inactivity: A Systematic Review and Meta-Analysis. BMC Geriatr..

[31] Medicine I. (2002). Dietary Reference Intakes for Energy, Carbohydrate, Fiber, Fat, Fatty Acids, Cholesterol, Protein, and Amino Acids.

[32] Dimeji I.Y., Abass K.S., Audu N.M., Ayodeji A.S. (2025). L-Arginine and Immune Modulation: A Pharmacological Perspective on Inflammation and Autoimmune Disorders. Eur. J. Pharmacol..

[33] Wu G., Meininger C.J., McNeal C.J., Bazer F.W., Rhoads J.M. (2021). Role of L-Arginine in Nitric Oxide Synthesis and Health in Humans. Adv. Exp. Med. Biol..

[34] Andrabi S.M., Sharma N.S., Karan A., Shatil Shahriar S.M., Cordon B., Ma B., Xie J., Andrabi S.M., Sharma N.S., Karan A. (2023). Nitric Oxide: Physiological Functions, Delivery, and Biomedical Applications. Adv. Sci..

[35] Peranzoni E., Marigo I., Dolcetti L., Ugel S., Sonda N., Taschin E., Mantelli B., Bronte V., Zanovello P. (2007). Role of Arginine Metabolism in Immunity and Immunopathology. Immunobiology.

[36] Witte M.B., Barbul A. (2002). Role of Nitric Oxide in Wound Repair. Am. J. Surg..

[37] Loehe F., Bruns C.J., Nitsch S.M., Angele M.K. (2007). The Role of L-Arginine Following Trauma and Blood Loss. Curr. Opin. Clin. Nutr. Metab. Care.

[38] Savustianenko A.V. (2018). L-Arginine Accelerates Wound Healing: New Mechanisms and Clinical Trial Data. TRAUMA.

[39] Karoń Ł., Zygmunt A.E., Karoń K., Grabowski W., Drapała G., Pedrycz E., Pedrycz D. (2024). L-Arginine Supplementation in Endurance Athletes: A Systematic Review of Recovery Mechanisms and Performance Enhancement. Qual. Sport.

[40] Curi R., Newsholme P., Marzuca-Nassr G.N., Takahashi H.K., Hirabara S.M., Cruzat V., Krause M., De Bittencourt P.I.H. (2016). Regulatory Principles in Metabolism-Then and Now. Biochem. J..

[41] Curi R., Lagranha C.J., Doi S.Q., Sellitti D.F., Procopio J., Pithon-Curi T.C., Corless M., Newsholme P. (2005). Molecular Mechanisms of Glutamine Action. J. Cell. Physiol..

[42] Cruzat V.F., Pantaleão L.C., Donato J., de Bittencourt P.I.H., Tirapegui J. (2014). Oral Supplementations with Free and Dipeptide Forms of L-Glutamine in Endotoxemic Mice: Effects on Muscle Glutamine-Glutathione Axis and Heat Shock Proteins. J. Nutr. Biochem..

[43] Warburg O. (1956). On the Origin of Cancer Cells. Science (1979).

[44] Yoo H.C., Yu Y.C., Sung Y., Han J.M. (2020). Glutamine Reliance in Cell Metabolism. Exp. Mol. Med..

[45] Newsholme P. (2001). Why Is L-Glutamine Metabolism Important to Cells of the Immune System in Health, Postinjury, Surgery or Infection?. J. Nutr..

[46] Wischmeyer P.E. (2003). Clinical Applications of L-Glutamine: Past, Present, and Future. Nutr. Clin. Pract..

[47] Hesterberg R.S., Cleveland J.L., Epling-Burnette P.K. (2018). Role of Polyamines in Immune Cell Functions. Med. Sci..

[48] Curi R., Lagranha C.J., Doi S.Q., Sellitti D.F., Procopio J., Pithon-Curi T.C. (2005). Glutamine-Dependent Changes in Gene Expression and Protein Activity. Cell Biochem. Funct..

[49] Hiscock N., Petersen E.W., Krzywkowski K., Boza J., Halkjaer-Kristensen J., Pedersen B.K. (2003). Glutamine Supplementation Further Enhances Exercise-Induced Plasma IL-6. J. Appl. Physiol. (1985).

[50] Roth E., Oehler R., Manhart N., Exner R., Wessner B., Strasser E., Spittler A. (2002). Regulative Potential of Glutamine—Relation to Glutathione Metabolism. Nutrition.

[51] Nan D., Yao W., Huang L., Liu R., Chen X., Xia W., Sheng H., Zhang H., Liang X., Lu Y. (2025). Glutamine and Cancer: Metabolism, Immune Microenvironment, and Therapeutic Targets. Cell Commun. Signal..

[52] Nelson V.L., Nguyen H.C.B., Garcìa-Cañaveras J.C., Briggs E.R., Ho W.Y., Dispirito J.R., Marinis J.M., Hill D.A., Lazar M.A. (2018). PPARγ Is a Nexus Controlling Alternative Activation of Macrophages via Glutamine Metabolism. Genes Dev..

[53] Liu P.S., Wang H., Li X., Chao T., Teav T., Christen S., DI Conza G., Cheng W.C., Chou C.H., Vavakova M. (2017). α-Ketoglutarate Orchestrates Macrophage Activation through Metabolic and Epigenetic Reprogramming. Nat. Immunol..

[54] Jayarajan S., Daly J.M. (2011). The Relationships of Nutrients, Routes of Delivery, and Immunocompetence. Surg. Clin. N. Am..

[55] Clerc I., Abba Moussa D., Vahlas Z., Tardito S., Oburoglu L., Hope T.J., Sitbon M., Dardalhon V., Mongellaz C., Taylor N. (2019). Entry of Glucose- and Glutamine-Derived Carbons into the Citric Acid Cycle Supports Early Steps of HIV-1 Infection in CD4 T Cells. Nat. Metab..

[56] El Ansari R., McIntyre A., Craze M.L., Ellis I.O., Rakha E.A., Green A.R. (2018). Altered Glutamine Metabolism in Breast Cancer; Subtype Dependencies and Alternative Adaptations. Histopathology.

[57] Ren W., Liu G., Yin J., Tan B., Wu G., Bazer F.W., Peng Y., Yin Y. (2017). Amino-Acid Transporters in T-Cell Activation and Differentiation. Cell Death Dis..

[58] Janakiram N.B., Mohammed A., Madka V., Kumar G., Rao C.V. (2016). Prevention and Treatment of Cancers by Immune Modulating Nutrients. Mol. Nutr. Food Res..

[59] Jiang S., Yan W., Wang S.E. (2019). MicroRNA Let-7 in B Lymphocyte Activation. Aging.

[60] Morikawa N., Tachibana M., Ago Y., Goda H., Sakurai F., Mizuguchi H. (2018). LY341495, an MGluR2/3 Antagonist, Regulates the Immunosuppressive Function of Myeloid-Derived Suppressor Cells and Inhibits Melanoma Tumor Growth. Biol. Pharm. Bull..

[61] Oh M.H., Sun I.H., Zhao L., Leone R.D., Sun I.M., Xu W., Collins S.L., Tam A.J., Blosser R.L., Patel C.H. (2020). Targeting Glutamine Metabolism Enhances Tumor-Specific Immunity by Modulating Suppressive Myeloid Cells. J. Clin. Investig..

[62] Jobin C., Hellerbrand C., Licato L.L., Brenner D.A., Sartor R.B. (1998). Mediation by NF-Kappa B of Cytokine Induced Expression of Intercellular Adhesion Molecule 1 (ICAM-1) in an Intestinal Epithelial Cell Line, a Process Blocked by Proteasome Inhibitors. Gut.

[63] Coëffier M., Claeyssens S., Hecketsweiler B., Lavoinne A., Ducrotté P., Déchelotte P. (2003). Enteral Glutamine Stimulates Protein Synthesis and Decreases Ubiquitin MRNA Level in Human Gut Mucosa. Am. J. Physiol. Gastrointest. Liver Physiol..

[64] Coëffier M., Miralles-Barrachina O., Le Pessot F., Lalaude O., Daveau M., Lavoinne A., Lerebours E., Déchelotte P. (2001). Influence of Glutamine on Cytokine Production by Human Gut in Vitro. Cytokine.

[65] Marian M.J. (2017). Dietary Supplements Commonly Used by Cancer Survivors: Are There Any Benefits?. Nutr. Clin. Pract..

[66] Sayles C., Hickerson S.C., Bhat R.R., Hall J., Garey K.W., Trivedi M.V. (2016). Oral Glutamine in Preventing Treatment-Related Mucositis in Adult Patients With Cancer: A Systematic Review. Nutr. Clin. Pract..

[67] Daniele B., Perrone F., Gallo C., Pignata S., De Martino S., De Vivo R., Barletta E., Tambaro R., Abbiati R., D’Agostino L. (2001). Oral Glutamine in the Prevention of Fluorouracil Induced Intestinal Toxicity: A Double Blind, Placebo Controlled, Randomised Trial. Gut.

[68] Fürst P., Alteheld B., Stehle P. (2004). Why Should a Single Nutrient—Glutamine—Improve Outcome?: The Remarkable Story of Glutamine Dipeptides. Clin. Nutr. Suppl..

[69] van Zanten A.R.H., Dhaliwal R., Garrel D., Heyland D.K. (2015). Enteral Glutamine Supplementation in Critically Ill Patients: A Systematic Review and Meta-Analysis. Crit. Care.

[70] Bodur M., Yilmaz B., Agagunduz D., Ozogul Y. (2025). Immunomodulatory Effects of Omega-3 Fatty Acids: Mechanistic Insights and Health Implications. Mol. Nutr. Food Res..

[71] Gladine C., Mazur A. (2014). Nutrigenomic Effects of Omega-3 Fatty Acids. Lipid Technol..

[72] Vanden Heuvel J.P. (2012). Nutrigenomics and Nutrigenetics of Ω3 Polyunsaturated Fatty Acids. Prog. Mol. Biol. Transl. Sci..

[73] Shahidi F., Miraliakbari H. (2005). Omega-3 Fatty Acids in Health and Disease: Part 2--Health Effects of Omega-3 Fatty Acids in Autoimmune Diseases, Mental Health, and Gene Expression. J. Med. Food.

[74] Venter C., Eyerich S., Sarin T., Klatt K.C. (2020). Nutrition and the Immune System: A Complicated Tango. Nutrients.

[75] Schoeniger A., Fuhrmann H., Schumann J. (2016). LPS- or Pseudomonas Aeruginosa-Mediated Activation of the Macrophage TLR4 Signaling Cascade Depends on Membrane Lipid Composition. PeerJ.

[76] Fan Y.-Y., Ly L.H., Barhoumi R., McMurray D.N., Chapkin R.S. (2004). Dietary Docosahexaenoic Acid Suppresses T Cell Protein Kinase C Theta Lipid Raft Recruitment and IL-2 Production. J. Immunol..

[77] Allam-Ndoul B., Guénard F., Barbier O., Vohl M.C. (2016). Effect of N-3 Fatty Acids on the Expression of Inflammatory Genes in THP-1 Macrophages. Lipids Health Dis..

[78] Jin J., Lu Z., Li Y., Cowart L.A., Lopes-Virella M.F., Huang Y. (2018). Docosahexaenoic Acid Antagonizes the Boosting Effect of Palmitic Acid on LPS Inflammatory Signaling by Inhibiting Gene Transcription and Ceramide Synthesis. PLoS ONE.

[79] Liu Y., Chen L.Y., Sokolowska M., Eberlein M., Alsaaty S., Martinez-Anton A., Logun C., Qi H.Y., Shelhamer J.H. (2014). The Fish Oil Ingredient, Docosahexaenoic Acid, Activates Cytosolic Phospholipase A_2_ via GPR120 Receptor to Produce Prostaglandin E_2_ and Plays an Anti-Inflammatory Role in Macrophages. Immunology.

[80] Calder P.C. (2013). Omega-3 polyunsaturated fatty acids and inflammatory processes: Nutritrion or pharmacology?. Br. J. Clin. Pharmacol..

[81] Serhan C.N. (2014). Pro-Resolving Lipid Mediators Are Leads for Resolution Physiology. Nature.

[82] Bannenberg G., Serhan C.N. (2010). Specialized Pro-Resolving Lipid Mediators in the Inflammatory Response: An Update. Biochim. Biophys. Acta (BBA)—Mol. Cell Biol. Lipids.

[83] Gutiérrez S., Svahn S.L., Johansson M.E. (2019). Effects of Omega-3 Fatty Acids on Immune Cells. Int. J. Mol. Sci..

[84] Whelan J., Gowdy K.M., Shaikh S.R. (2016). N-3 Polyunsaturated Fatty Acids Modulate B Cell Activity in Pre-Clinical Models: Implications for the Immune Response to Infections. Eur. J. Pharmacol..

[85] Wang H., Hao Q., Li Q.R., Yan X.W., Ye S., Li Y.S., Li N., Li J.S. (2007). Omega-3 Polyunsaturated Fatty Acids Affect Lipopolysaccharide-Induced Maturation of Dendritic Cells through Mitogen-Activated Protein Kinases P38. Nutrition.

[86] Chang H.Y., Lee H.N., Kim W., Surh Y.J. (2015). Docosahexaenoic Acid Induces M2 Macrophage Polarization through Peroxisome Proliferator-Activated Receptor γ Activation. Life Sci..

[87] Williams-Bey Y., Boularan C., Vural A., Huang N.N., Hwang I.Y., Shan-Shi C., Kehrl J.H. (2014). Omega-3 Free Fatty Acids Suppress Macrophage Inflammasome Activation by Inhibiting NF-ΚB Activation and Enhancing Autophagy. PLoS ONE.

[88] Yan Y., Jiang W., Spinetti T., Tardivel A., Castillo R., Bourquin C., Guarda G., Tian Z., Tschopp J., Zhou R. (2013). Omega-3 Fatty Acids Prevent Inflammation and Metabolic Disorder through Inhibition of NLRP3 Inflammasome Activation. Immunity.

[89] Vemulapalli V., Shirwaikar Thomas A. (2025). The Role of Vitamin D in Gastrointestinal Homeostasis and Gut Inflammation. Int. J. Mol. Sci..

[90] Fekete M., Lehoczki A., Szappanos Á., Zábó V., Kaposvári C., Horváth A., Farkas Á., Fazekas-Pongor V., Major D., Lipécz Á. (2025). Vitamin D and Colorectal Cancer Prevention: Immunological Mechanisms, Inflammatory Pathways, and Nutritional Implications. Nutrients.

[91] Chen Y., Zhang J., Ge X., Du J., Deb D.K., Li Y.C. (2013). Vitamin D Receptor Inhibits Nuclear Factor ΚB Activation by Interacting with IκB Kinase β Protein. J. Biol. Chem..

[92] Jeon S.M., Shin E.A. (2018). Exploring Vitamin D Metabolism and Function in Cancer. Exp. Mol. Med..

[93] Ghaseminejad-Raeini A., Ghaderi A., Sharafi A., Nematollahi-Sani B., Moossavi M., Derakhshani A., Sarab G.A. (2023). Immunomodulatory Actions of Vitamin D in Various Immune-Related Disorders: A Comprehensive Review. Front. Immunol..

[94] Martens P.J., Gysemans C., Verstuyf A., Mathieu C. (2020). Vitamin D’s Effect on Immune Function. Nutrients.

[95] Hafkamp F.M.J., Taanman-Kueter E.W.M., van Capel T.M.M., Kormelink T.G., de Jong E.C. (2022). Vitamin D3 Priming of Dendritic Cells Shifts Human Neutrophil-Dependent Th17 Cell Development to Regulatory T Cells. Front. Immunol..

[96] Cartes-Velásquez R., Vera A., Torres-Quevedo R., Medrano-Díaz J., Pérez A., Muñoz C., Carrillo-Bestagno H., Nova-Lamperti E. (2024). The Immunomodulatory Role of Vitamin D in Regulating the Th17/Treg Balance and Epithelial–Mesenchymal Transition: A Hypothesis for Gallbladder Cancer. Nutrients.

[97] Rolf L., Muris A.H., Hupperts R., Damoiseaux J. (2014). Vitamin D Effects on B Cell Function in Autoimmunity. Ann. N. Y. Acad. Sci..

[98] Dankers W., Colin E.M., van Hamburg J.P., Lubberts E. (2017). Vitamin D in Autoimmunity: Molecular Mechanisms and Therapeutic Potential. Front. Immunol..

[99] Sun J., Zhang Y.G. (2022). Vitamin D Receptor Influences Intestinal Barriers in Health and Disease. Cells.

[100] Chatterjee I., Zhang Y., Zhang J., Lu R., Xia Y., Sun J. (2021). Overexpression of Vitamin D Receptor in Intestinal Epithelia Protects Against Colitis via Upregulating Tight Junction Protein Claudin 15. J. Crohns Colitis.

[101] Zhang Y., Garrett S., Carroll R.E., Xia Y., Sun J. (2022). Vitamin D Receptor Upregulates Tight Junction Protein Claudin-5 against Colitis-Associated Tumorigenesis. Mucosal Immunol..

[102] Bellerba F., Muzio V., Gnagnarella P., Facciotti F., Chiocca S., Bossi P., Cortinovis D., Chiaradonna F., Serrano D., Raimondi S. (2021). The Association between Vitamin D and Gut Microbiota: A Systematic Review of Human Studies. Nutrients.

[103] Bartolacci A., Stocchi F., Stocchi V., Zeppa S.D. (2025). The Crucial Role of Vitamin D in Regulating Gut Microbiota in Inflammatory Bowel Disease. Recent Prog. Nutr..

[104] Vernia F., Valvano M., Longo S., Cesaro N., Viscido A., Latella G. (2022). Vitamin D in Inflammatory Bowel Diseases. Mechanisms of Action and Therapeutic Implications. Nutrients.

[105] Bashir M., Prietl B., Tauschmann M., Mautner S.I., Kump P.K., Treiber G., Wurm P., Gorkiewicz G., Högenauer C., Pieber T.R. (2016). Effects of High Doses of Vitamin D3 on Mucosa-Associated Gut Microbiome Vary between Regions of the Human Gastrointestinal Tract. Eur. J. Nutr..

[106] Ananthakrishnan A.N., Cagan A., Gainer V.S., Cheng S.C., Cai T., Szolovits P., Shaw S.Y., Churchill S., Karlson E.W., Murphy S.N. (2014). Higher Plasma Vitamin D Is Associated with Reduced Risk of Clostridium Difficile Infection in Patients with Inflammatory Bowel Diseases. Aliment. Pharmacol. Ther..

[107] Popescu G.A., Minca D.G., Jafal N.M., Toma C.V., Alexandrescu S.T., Costea R.V., Vasilescu C. (2025). Multimodal Prehabilitation in Major Abdominal Surgery—Rationale, Modalities, Results and Limitations. Medicina.

[108] Chris Ugbolue U., Percy Marshall R., Alizadeh Pahlavani H., Khang Duy Ricky Le C. (2025). Integration of Resistance Exercise into a Multimodal Approach to Prehabilitation for Patients with Sarcopenia Prior to Surgery: A Narrative Review. Front. Rehabil. Sci..

[109] Suárez-Alcázar M.P., Folch Ayora A., Muriach M., Recacha-Ponce P., Garcia-Roca M.E., Coret-Franco A., Pastor-Mora J.C., Salas-Medina P., Collado-Boira E.J. (2025). Multimodal Prehabilitation in Colorectal Cancer: Improving Fitness, Lifestyle, and Post-Surgery Outcomes. Healthcare.

[110] Egan B., Sharples A.P. (2023). Molecular responses to acute exercise and their relevance for adaptations in skeletal muscle to exercise training. Physiol. Rev..

[111] Liu H., Wang S., Wang J., Guo X., Song Y., Fu K., Gao Z., Liu D., He W., Yang L.L. (2025). Energy Metabolism in Health and Diseases. Signal Transduct. Target. Ther..

[112] Smith J.A.B., Murach K.A., Dyar K.A., Zierath J.R. (2023). Exercise Metabolism and Adaptation in Skeletal Muscle. Nat. Rev. Mol. Cell Biol..

[113] Damanti S., Senini E., De Lorenzo R., Merolla A., Santoro S., Festorazzi C., Messina M., Vitali G., Sciorati C., Rovere-Querini P. (2024). Acute Sarcopenia: Mechanisms and Management. Nutrients.

[114] Tezze C., Sandri M., Tessari P. (2023). Anabolic Resistance in the Pathogenesis of Sarcopenia in the Elderly: Role of Nutrition and Exercise in Young and Old People. Nutrients.

[115] Feng L., Li B., Yong S.S., Wu X., Tian Z. (2024). Exercise and Nutrition Benefit Skeletal Muscle: From Influence Factor and Intervention Strategy to Molecular Mechanism. Sports Med. Health Sci..

[116] Qian L., Zhu Y., Deng C., Liang Z., Chen J., Chen Y., Wang X., Liu Y., Tian Y., Yang Y. (2024). Peroxisome Proliferator-Activated Receptor Gamma Coactivator-1 (PGC-1) Family in Physiological and Pathophysiological Process and Diseases. Signal Transduct. Target. Ther..

[117] Wang J., Jia D., Zhang Z., Wang D. (2025). Exerkines and Sarcopenia: Unveiling the Mechanism Behind Exercise-Induced Mitochondrial Homeostasis. Metabolites.

[118] Novelli G., Calcaterra G., Casciani F., Pecorelli S., Mehta J.L. (2024). “Exerkines”: A Comprehensive Term for the Factors Produced in Response to Exercise. Biomedicines.

[119] Pervin S., Reddy S.T., Singh R. (2021). Novel Roles of Follistatin/Myostatin in Transforming Growth Factor-β Signaling and Adipose Browning: Potential for Therapeutic Intervention in Obesity Related Metabolic Disorders. Front. Endocrinol..

[120] Wetzlich B., Nyakundi B.B., Yang J. (2024). Therapeutic Applications and Challenges in Myostatin Inhibition for Enhanced Skeletal Muscle Mass and Functions. Mol. Cell. Biochem..

[121] Shen S., Liao Q., Chen X., Peng C., Lin L. (2022). The Role of Irisin in Metabolic Flexibility: Beyond Adipose Tissue Browning. Drug Discov. Today.

[122] Yoshida T., Delafontaine P. (2020). Mechanisms of IGF-1-Mediated Regulation of Skeletal Muscle Hypertrophy and Atrophy. Cells.

[123] Feng L., Li B., Xi Y., Cai M., Tian Z. (2022). Aerobic Exercise and Resistance Exercise Alleviate Skeletal Muscle Atrophy through IGF-1/IGF-1R-PI3K/Akt Pathway in Mice with Myocardial Infarction. Am. J. Physiol. Cell Physiol..

[124] Nara H., Watanabe R. (2021). Anti-Inflammatory Effect of Muscle-Derived Interleukin-6 and Its Involvement in Lipid Metabolism. Int J. Mol. Sci..

[125] Docherty S., Harley R., McAuley J.J., Crowe L.A.N., Pedret C., Kirwan P.D., Siebert S., Millar N.L. (2022). The Effect of Exercise on Cytokines: Implications for Musculoskeletal Health: A Narrative Review. BMC Sports Sci. Med. Rehabil..

[126] Novick D., Chong W.P., Małkowska P., Sawczuk M. (2023). Cytokines as Biomarkers for Evaluating Physical Exercise in Trained and Non-Trained Individuals: A Narrative Review. Int. J. Mol. Sci..

[127] Li M., Jiang P., Wei S., Wang J., Li C. (2023). The Role of Macrophages-Mediated Communications among Cell Compositions of Tumor Microenvironment in Cancer Progression. Front. Immunol..

[128] Yu X., Pei W., Li B., Sun S., Li W., Wu Q. (2025). Immunosenescence, Physical Exercise, and Their Implications in Tumor Immunity and Immunotherapy. Int. J. Biol. Sci..

[129] Hu T., Liu C.H., Lei M., Zeng Q., Li L., Tang H., Zhang N. (2024). Metabolic Regulation of the Immune System in Health and Diseases: Mechanisms and Interventions. Signal Transduct. Target. Ther..

[130] Ferrer M., Anthony T.G., Ayres J.S., Biffi G., Brown J.C., Caan B.J., Cespedes Feliciano E.M., Coll A.P., Dunne R.F., Goncalves M.D. (2023). Cachexia: A Systemic Consequence of Progressive, Unresolved Disease. Cell.

[131] Lu X., Chen Y., Shi Y., Shi Y., Su X., Chen P., Wu D., Shi H. (2025). Exercise and Exerkines: Mechanisms and Roles in Anti-Aging and Disease Prevention. Exp. Gerontol..

[132] Walzik D., Wences Chirino T.Y., Zimmer P., Joisten N. (2024). Molecular Insights of Exercise Therapy in Disease Prevention and Treatment. Signal Transduct. Target. Ther..

[133] Guo X., Si Y., Liu H., Yu L. (2024). Effects of Aerobic Exercise on Cardiopulmonary Function in Postoperative Patients with Congenital Heart Disease: A Meta-Analysis. Rev. Cardiovasc. Med..

[134] Parker T., Brealey D., Dyson A., Singer M. (2019). Optimising Organ Perfusion in the High-Risk Surgical and Critical Care Patient: A Narrative Review. Br. J. Anaesth..

[135] Prado C.M., Landi F., Chew S.T.H., Atherton P.J., Molinger J., Ruck T., Gonzalez M.C. (2022). Advances in Muscle Health and Nutrition: A Toolkit for Healthcare Professionals. Clin. Nutr..

[136] Hong A.R., Hong S.M., Shin Y.A. (2014). Effects of Resistance Training on Muscle Strength, Endurance, and Motor Unit According to Ciliary Neurotrophic Factor Polymorphism in Male College Students. J. Sports Sci. Med..

[137] Prado C.M., Purcell S.A., Alish C., Pereira S.L., Deutz N.E., Heyland D.K., Goodpaster B.H., Tappenden K.A., Heymsfield S.B. (2018). Implications of Low Muscle Mass across the Continuum of Care: A Narrative Review. Ann. Med..

[138] Nozoe M., Kubo H., Yamamoto M., Ikeji R., Seike H., Majima K., Shimada S. (2024). Muscle Weakness Is More Strongly Associated with Functional Outcomes in Patients with Stroke than Sarcopenia or Muscle Wasting: An Observational Study. Aging Clin. Exp. Res..

[139] Hughes D.C., Ellefsen S., Baar K. (2018). Adaptations to Endurance and Strength Training. Cold Spring Harb. Perspect. Med..

[140] Riviati N., Indra B. (2023). Relationship between Muscle Mass and Muscle Strength with Physical Performance in Older Adults: A Systematic Review. SAGE Open Med..

[141] Bojesen R.D., Dalton S.O., Skou S.T., Jørgensen L.B., Walker L.R., Eriksen J.R., Grube C., Justesen T.F., Johansen C., Slooter G. (2023). Preoperative Multimodal Prehabilitation before Elective Colorectal Cancer Surgery in Patients with WHO Performance Status I or II: Randomized Clinical Trial. BJS Open.

[142] Molenaar C.J.L., van Rooijen S.J., Fokkenrood H.J.P., Roumen R.M.H., Janssen L., Slooter G.D. (2023). Prehabilitation versus No Prehabilitation to Improve Functional Capacity, Reduce Postoperative Complications and Improve Quality of Life in Colorectal Cancer Surgery. Cochrane Database Syst. Rev..

[143] Koh F.H.X., Yik V., Chin S.E., Kok S.S.X., Lee H.B., Tong C., Tay P., Chean E., Lam Y.E., Mah S.M. (2024). Evaluating the Impact of Multimodal Prehabilitation with High Protein Oral Nutritional Supplementation (HP ONS) with Beta-Hydroxy Beta-Methylbutyrate (HMB) on Sarcopenic Surgical Patients—Interim Analysis of the HEROS Study. Nutrients.

[144] Shanmugasundaram Prema S., Ganapathy D., Shanmugamprema D. (2025). Prehabilitation Strategies: Enhancing Surgical Resilience with a Focus on Nutritional Optimization and Multimodal Interventions. Adv. Nutr..

[145] Gillis C., Ljungqvist O., Carli F. (2022). Prehabilitation, Enhanced Recovery after Surgery, or Both? A Narrative Review. Br. J. Anaesth..

[146] Hirst N., McBride K., Steffens D. (2024). Psychological Interventions in Prehabilitation Randomized Controlled Trials for Patients Undergoing Cancer Surgery: Sufficient or Suboptimal?. Ann. Surg. Oncol..

[147] Levett D.Z.H., Grimmett C. (2019). Psychological Factors, Prehabilitation and Surgical Outcomes: Evidence and Future Directions. Anaesthesia.

[148] Powell R., Scott N.W., Manyande A., Bruce J., Vögele C., Byrne-Davis L.M.T., Unsworth M., Osmer C., Johnston M. (2016). Psychological Preparation and Postoperative Outcomes for Adults Undergoing Surgery under General Anaesthesia. Cochrane Database Syst. Rev..

[149] Periañez C.A.H., Castillo-Díaz M.A. (2025). Preoperative Psychological Distress and Acute Postoperative Pain among Abdominal Surgery Patients. J. Psychosom. Res..

[150] Baagil H., Baagil H., Gerbershagen M.U. (2023). Preoperative Anxiety Impact on Anesthetic and Analgesic Use. Medicina.

[151] Ni K., Zhu J., Ma Z. (2023). Preoperative Anxiety and Postoperative Adverse Events: A Narrative Overview. Anesthesiol. Perioper. Sci..

[152] Gillis C., Fenton T.R., Sajobi T.T., Minnella E.M., Awasthi R., Loiselle S.È., Liberman A.S., Stein B., Charlebois P., Carli F. (2019). Trimodal Prehabilitation for Colorectal Surgery Attenuates Post-Surgical Losses in Lean Body Mass: A Pooled Analysis of Randomized Controlled Trials. Clin. Nutr..

[153] Anghel T., Melania B.L., Costea I., Albai O., Marinca A., Levai C.M., Hogea L.M. (2025). Review of Psychological Interventions in Oncology: Current Trends and Future Directions. Medicina.

[154] Grimmett C., Heneka N., Chambers S. (2022). Psychological Interventions Prior to Cancer Surgery: A Review of Reviews. Curr. Anesthesiol. Rep..

[155] Rucińska M., Osowiecka K. (2022). Prehabilitation as an Extra Approach to Usual Care for Cancer Patients. Nowotwory. J. Oncol..

[156] Nasir M., Scott E.J., Westermann R.C. (2023). Pain Catastrophizing, Kinesiophobia, Stress, Depression, and Poor Resiliency Are Associated With Pain and Dysfunction in the Hip Preservation Population. Iowa Orthop. J..

[157] Sharif-Nia H., Nazari R., Hajihosseini F., Froelicher E.S., Osborne J.W., Taebbi S., Nowrozi P. (2025). The Relationship of Fear of Pain, Pain Anxiety, and Fear-Avoidance Beliefs with Perceived Stress in Surgical Patients with Postoperative Kinesiophobia. BMC Psychol..

[158] Biswal B., Gandhi Y., Singla D.R., Velleman R., Zhou B., Fernandes L., Patel V., Prina M., Sequeira M., Garg A. (2024). Interventions for Improving Adherence to Psychological Treatments for Common Mental Disorders: A Systematic Review. Camb. Prism. Glob. Ment. Health.

[159] Antoni M.H., Moreno P.I., Penedo F.J. (2022). Stress Management Interventions to Facilitate Psychological and Physiological Adaptation and Optimal Health Outcomes in Cancer Patients and Survivors. Annu. Rev. Psychol..

[160] Lanini I., Amass T., Calabrisotto C.S., Fabbri S., Falsini S., Adembri C., Di Filippo A., Romagnoli S., Villa G. (2022). The Influence of Psychological Interventions on Surgical Outcomes: A Systematic Review. J. Anesth. Analg. Crit. Care.

[161] Garssen B., Boomsma M.F., Beelen R.H.J. (2010). Psychological Factors in Immunomodulation Induced by Cancer Surgery: A Review. Biol. Psychol..

[162] Herman J.P., McKlveen J.M., Ghosal S., Kopp B., Wulsin A., Makinson R., Scheimann J., Myers B. (2016). Regulation of the Hypothalamic-Pituitary-Adrenocortical Stress Response. Compr. Physiol..

[163] James K.A., Stromin J.I., Steenkamp N., Combrinck M.I. (2023). Understanding the Relationships between Physiological and Psychosocial Stress, Cortisol and Cognition. Front. Endocrinol..

[164] Gouin J.P., Kiecolt-Glaser J.K. (2011). The Impact of Psychological Stress on Wound Healing: Methods and Mechanisms. Immunol. Allergy Clin. N. Am..

[165] Balakin E., Yurku K., Ivanov M., Izotov A., Nakhod V., Pustovoyt V. (2025). Regulation of Stress-Induced Immunosuppression in the Context of Neuroendocrine, Cytokine, and Cellular Processes. Biology.

[166] Frasier K., Li V., Christoforides S., Daly K., Loperfito A., Stech K., Dragovic M., Frasier K., Li V., Christoforides S. (2024). The Impact of Psychosocial Influences on Chronic Wound Healing. Open J. Med. Psychol..

[167] Vargas-Uricoechea H., Castellanos-Pinedo A., Urrego-Noguera K., Vargas-Sierra H.D., Pinzón-Fernández M.V., Barceló-Martínez E., Ramírez-Giraldo A.F. (2024). Mindfulness-Based Interventions and the Hypothalamic–Pituitary–Adrenal Axis: A Systematic Review. Neurol. Int..

[168] Rajasundaram S., Rahman R.P., Woolf B., Zhao S.S., Gill D. (2022). Morning Cortisol and Circulating Inflammatory Cytokine Levels: A Mendelian Randomisation Study. Genes.

[169] Wunderle C., Martin E., Wittig A., Tribolet P., Lutz T.A., Köster-Hegmann C., Stanga Z., Mueller B., Schuetz P. (2025). Comparison of the Inflammatory Biomarkers IL- 6, TNF-α, and CRP to Predict the Effect of Nutritional Therapy on Mortality in Medical Patients at Risk of Malnutrition: A Secondary Analysis of the Randomized Clinical Trial EFFORT. J. Inflamm..

[170] Hajong R., Newme K., Nath C.K., Moirangthem T., Dhal M.R., Pala S. (2021). Role of Serum C-Reactive Protein and Interleukin-6 as a Predictor of Intra-Abdominal and Surgical Site Infections after Elective Abdominal Surgery. J. Family Med. Prim. Care.

[171] Nieman D.C., Wentz L.M. (2019). The Compelling Link between Physical Activity and the Body’s Defense System. J. Sport Health Sci..

[172] Lassale C., Batty G.D., Baghdadli A., Jacka F., Sánchez-Villegas A., Kivimäki M., Akbaraly T. (2019). Healthy Dietary Indices and Risk of Depressive Outcomes: A Systematic Review and Meta-Analysis of Observational Studies. Mol. Psychiatry.

[173] Marshall G.D. (2011). The Adverse Effects of Psychological Stress on Immunoregulatory Balance: Applications to Human Inflammatory Diseases. Immunol. Allergy Clin. N. Am..

[174] Shchaslyvyi A.Y., Antonenko S.V., Telegeev G.D. (2024). Comprehensive Review of Chronic Stress Pathways and the Efficacy of Behavioral Stress Reduction Programs (BSRPs) in Managing Diseases. Int. J. Environ. Res. Public Health.

[175] De Assis L.V.M., Kramer A. (2024). Circadian de(Regulation) in Physiology: Implications for Disease and Treatment. Genes Dev..

[176] Singh K.K., Ghosh S., Bhola A., Verma P., Amist A.D., Sharma H., Sachdeva P., Sinha J.K. (2024). Sleep and Immune System Crosstalk: Implications for Inflammatory Homeostasis and Disease Pathogenesis. Ann. Neurosci..

[177] Xu N., Zhang J.-X., Zhang J.-J., Huang Z., Mao L.-C., Zhang Z.-Y., Jin W.-D. (2025). The Prognostic Value of the Neutrophil-to-Lymphocyte Ratio (NLR) and Platelet-to-Lymphocyte Ratio (PLR) in Colorectal Cancer and Colorectal Anastomotic Leakage Patients: A Retrospective Study. BMC Surg..

[178] Li Q., Geng S., Luo H., Wang W., Mo Y.-Q., Luo Q., Wang L., Song G.-B., Sheng J.-P., Xu B. (2024). Signaling Pathways Involved in Colorectal Cancer: Pathogenesis and Targeted Therapy. Signal Transduct. Target. Ther..

[179] Allahyari A., Fallah F., Taqanaki P.B., Tafti S.P., Vakilzadeh M.M., Nodeh M.M., Kamandi M., Noferesti A. (2024). Evaluating the Neutrophil-to-Lymphocyte Ratio (NLR) and Platelet-to-Lymphocyte Ratio (PLR) as Prognostic and Treatment Response Biomarkers in Stage IV Colorectal Cancer Patients. Oncol. Clin. Pract..

[180] Bausys A., Kryzauskas M., Abeciunas V., Degutyte A.E., Bausys R., Strupas K., Poskus T. (2022). Prehabilitation in Modern Colorectal Cancer Surgery: A Comprehensive Review. Cancers.

[181] Xu S., Yin R., Zhu H., Gong Y., Zhu J., Li C., Xu Q. (2025). The Role of Exercise-Based Prehabilitation in Enhancing Surgical Outcomes for Patients with Digestive System Cancers: A Meta-Analysis. BMC Gastroenterol..

[182] Mcisaac D.I., Kidd G., Gillis C., Branje K., Al-Bayati M., Baxi A., Grudzinski A.L., Boland L., Veroniki A.A., Wolfe D. (2025). Relative Efficacy of Prehabilitation Interventions and Their Components: Systematic Review with Network and Component Network Meta-Analyses of Randomised Controlled Trials. BMJ.

[183] Delle Cave D. (2025). Advances in Molecular Mechanisms and Therapeutic Strategies in Colorectal Cancer: A New Era of Precision Medicine. Int. J. Mol. Sci..

[184] He S., Zhang S., Yao Y., Xu B., Niu Z., Liao F., Wu J., Song Q., Li M., Liu Z. (2022). Turbulence of Glutamine Metabolism in Pan-Cancer Prognosis and Immune Microenvironment. Front. Oncol..

[185] Li X., Zhu H., Sun W., Yang X., Nie Q., Fang X. (2021). Role of Glutamine and Its Metabolite Ammonia in Crosstalk of Cancer-Associated Fibroblasts and Cancer Cells. Cancer Cell Int..

[186] Zou W., Han Z., Wang Z., Liu Q. (2025). Targeting Glutamine Metabolism as a Potential Target for Cancer Treatment. J. Exp. Clin. Cancer Res..

[187] Qian L., Zhang F., Yin M., Lei Q. (2022). Cancer Metabolism and Dietary Interventions. Cancer Biol. Med..

[188] Ruban M., Pozhidaeva E., Bolotina L., Kaprin A. (2025). The Role of Diet and Nutrition in Cancer Development and Management: From Molecular Mechanisms to Personalized Interventions. Foods.

